# CXCL12 expression promotes esophageal squamous cell carcinoma proliferation and worsens the prognosis

**DOI:** 10.1186/s12885-016-2555-z

**Published:** 2016-07-21

**Authors:** Yusuke Uchi, Hiroya Takeuchi, Sachiko Matsuda, Yoshiro Saikawa, Hirofumi Kawakubo, Norihito Wada, Tsunehiro Takahashi, Rieko Nakamura, Kazumasa Fukuda, Tai Omori, Yuko Kitagawa

**Affiliations:** Department of Surgery, Keio University School of Medicine, 160-0016, 35 Shinanomachi, Shinjuku-ku, Tokyo, Japan

**Keywords:** Chemokine, CXCL12, Chemokine receptor, CXCR4, Esophageal squamous cell carcinoma

## Abstract

**Background:**

The chemokine CXCL12 and its corresponding receptor CXCR4 are key players in the development of several cancers. Therefore, we hypothesized that there is a functional causality between CXCL12 expression and tumor progression in patients with esophageal squamous cell carcinoma (ESCC).

**Methods:**

We performed an immunohistochemical analysis in 79 consecutive patients with ESCC. We performed in vitro and in vivo cell proliferation assays using ESCC cell lines and a newly established transfectant stably overexpressing CXCL12.

**Results:**

Immunohistochemistry revealed positive CXCR4 and CXCL12 expression in 48 (61 %) and 62 (78 %) patients, respectively. Additionally, the expression levels did not significantly correlate with any clinicopathological factors. The MIB-1 proliferation index was markedly higher in ESCC with a positive expression of CXCR4 or CXCL12. Positive CXCL12 expression was significantly correlated with lower recurrence-free survival (RFS, *p* = 0.02). Cox’s hazard models revealed CXCL12 expression as an independent predictive factor for recurrence. In vitro*,* CXCL12 exposure or overexpression enhanced ESCC proliferation; and AMD3100, a specific inhibitor of CXCR4, equally decreased proliferation irrespective of CXCL12 exposure or overexpression. In the mouse model, AMD3100 significantly decreased ESCC tumor size (*p* = 0.03).

**Conclusions:**

CXCL12 stimulates ESCC proliferation, and its expression levels are related to lower RFS in patients with ESCC. Our findings indicate that positive CXCL12 expression may be a useful marker for predicting the outcome in patients with ESCC and is a potentially new therapeutic target for ESCC.

## Background

Esophageal cancer is one of the most malignant solid tumors with a 5-year survival rate of <19 % [1]. Squamous cell carcinoma (SCC) is one of the two most common histologic types of esophageal cancer. The three main treatment options available for esophageal SCC (ESCC) are surgery, chemotherapy and radiotherapy. Studies evaluating the efficacy of combining these three modalities showed only a limited improvement in the prognosis [2–5].

A potential breakthrough may arise if the mechanism of ESCC proliferation can be clarified. Several studies reported that chemokines with lymphocyte chemoattractant capacities and chemokine receptors promote the progression of malignant tumors [6–10]. CXCR4, the receptor for chemokine CXCL12, was defined as the co-receptor used by the T-lymphotropic human immunodeficiency virus (HIV)-type 1 strains for cellular entry [11]. AMD3100, a bicyclam in which two cyclam rings are tethered by an aromatic bridge, selectively blocks CXCR4 and inhibits HIV replication [12]. Specifically, it blocks the interaction between gp120 (viral envelope glycoprotein) and CXCR4. The correlation was reported between the inhibitory effects of AMD3100 on HIV-1 replication, and CXCL12-mediated signal transduction (CXCL12-induced Ca^2+^ flux). AMD3100 is a highly specific CXCR4 antagonist inhibiting CXCL12-mediated Ca^2+^ flux in a number of cells expressing CXCR4, but has no inhibitory effect on chemokine induced signalling from other chemokine receptors [11]. AMD3100 interacts with CXCR4 extracellularly and mechanistically prevents the binding of CXCL12 to CXCR4 and thus downstream signalling of CXCR4. [11, 13]. AMD3100 is not toxic to host cells at concentrations up to 500 mM and the CXCL12/CXCR4 blockade efficiently decreases cancer cell proliferation [11, 14].

Although the relationship between CXCL12/CXCR4 expression levels and cell behaviour was described in a variety of malignant tumors [14–18], few have assessed this relationship for esophageal cancer. In this study, we analysed the relationship between CXCL12/CXCR4 expression and tumor proliferation in patients with ESCC. We further investigated the correlation between CXCL12/CXCR4 expression levels and clinicopathological features. Our in vitro and in vivo results show that CXCL12 promotes ESCC proliferation.

## Methods

### Patient selection

Patients were enrolled in the study if they met the following inclusion criteria: (1) pathologically confirmed ESCC after radical esophagectomy at Keio University Hospital between 1997 and 2007; (2) no history of radiotherapy or chemotherapy prior to surgery; (3) R0 resection and (4) complete follow-up information. Each patient enrolled in this study signed an informed consent form.

This study was approved by the Institutional Review Board at Keio University School of Medicine.

### Immunohistochemistry

Esophageal specimen tissues were obtained from surgical resection. Tumor samples were fixed with 10 % formalin in phosphate-buffered saline, embedded in paraffin and sectioned into 4-μm slices. The slides were deparaffinised in xylene and dehydrated in a graded ethanol series. The sections were incubated in Target Retrieval Solution PH6.0 (Dako, Glostrup, Denmark) for 10 min at 121 °C. The endogenous peroxidase activity was blocked by immersing the slides in 0.5 % periodic acid for 10 min at room temperature. After washing with water, the sections were treated with 4 % BlockAce (DS Pharma Biomedical, Osaka, Japan) for 30 min to block nonspecific reactions at 37 °C. The blocked sections were incubated with the primary diluted antibody at 4 °C overnight. The primary antibodies were diluted as follows: CXCL12 (MAB350, 1:200; R&D Systems, Minneapolis, USA) and CXCR4 (ab2074, 1:500; Abcam Inc., Cambridge, UK) were diluted in Can Get Signal Immunostain Solution A (Toyobo, Osaka, Japan), and Ki67 (SP6, 1:200; Thermo Fisher Scientific, Waltham, USA) was diluted in 4 % BlockAce. The sections were rinsed with Tris-buffered saline (TBS) followed by an incubation with ENVISION (Dako) for 30 min at room temperature. After the TBS washes, the sections were stained with a DAB kit (Dako), counterstained with hematoxylin and mounted. For each staining protocol, a negative control without the primary antibody was included. We evaluated CXCL12, CXCR4 and Ki67 expression on slides for viable ESCC cells belonging to the most invasive tumor lesion. We observed four different vision fields under high magnification (×400). To assess the immunoreactivity of CXCL12 and CXCR4, the sections were scored in terms of their proportion (score 0: −10 %, 1: 10–40 %, 2: 40–70 %, and 3: >70 %) and intensity (score 0: none, 1: weak, and 2: strong). The immunoreactive score (IRS) was defined as the product of the proportion and intensity scores. For the final statistical analysis, an IRS value of 0 was ranked as the negative expression and IRS values of 1–6 were ranked as positive expression. The MIB-1 proliferation index was calculated as the percentage of Ki67 positive cells. Two investigators (UY and TH) blinded to the clinicopathological factors assessed the immunohistochemistry.

### Animals

All animal experiments were executed according to Institutional Guidelines on Animal Experimentation at Keio University and were approved by The Keio University Institutional Animal Care and Use Committee. Female BALB/cA nude mice were purchased from Oriental Yeast Co., Ltd (Tokyo, Japan). The mice were maintained under specific pathogen-free conditions at Keio University Experimental Animal Center and fed sterile food and water. In each experiment, 10 mice aged 6 weeks were used. They were allocated to two groups and the weight prior to the intervention did not significantly differ among the groups (data not shown).

### Esophageal cell lines

We used eight established ESCC cell lines (TE-1, 4, 5, 6, 8, 9, 10, and 11) provided by Dr. Nishihira (Tohoku University, Miyagi, Japan). The identity of each cell line was confirmed by short tandem repeat analysis [10].

### RNA extraction and quantitative real-time RT-PCR

Total RNA from each ESCC cell line was extracted and analysed by quantitative real-time RT-PCR using the 7300 Real Time PCR system (Applied BioSystems, Carlsbad, CA), TaqMan Gene Expression Master Mix (Applied BioSystems) and ready-to-use CXCR4/CXCL12 primers (Assay ID: Hs00607978_m1 for CXCR4 and Hs00171022_m1 for CXCL12; Applied BioSystems). Glyceraldehyde-3-phosphate dehydrogenase was used as an internal control. We used human lymphocytes from a healthy donor (UY) as a positive control and distilled water without the template as a negative control. The relative quantity of CXCR4 and CXCL12 mRNA in ESCC cell lines was calculated using the ΔΔCt method. The expression levels of TE1 and TE4 were defined as 1 for the evaluation of CXCR4 and CXCL12 expression levels, respectively. All assays were performed in triplicate.

### Establishment of a stable CXCL12-overexpressing cell line

CXCL12 mRNA was extracted from a healthy volunteer’s (UY) lymphocytes. The full-length open reading frame was amplified and inserted into the plasmid vector pFLAG-CMV-4 (Sigma Aldrich, St. Louis, MO). The plasmids were transfected to TE4 cells using Lipofectamine 2000 Reagent (Invitrogen, Carlsbad, CA). When CXCL12 overexpression was confirmed by quantitative real-time RT-PCR, the stable transfectant was denoted TE4^CXCL12+^.

### In vitro proliferation assay

We examined the effect of CXCL12 on ESCC proliferation using water soluble tetrazolium (WST) salt. TE4 and TE4^CXCL12+^ (1 × 10^4^) were seeded in a 96-well plate (day 0) after overnight serum starvation. The following day, cells were exposed to different concentrations of CXCL12 (25 nM, R&D Systems) and/or AMD3100. Cell viability was measured using Cell Counting Kit-8 (Dojindo, Kumamoto, Japan) per the manufacturer’s instruction after 72 h. The assays were performed in triplicate. We defined the ‘proliferation index’ as the absorbance ratio of day 3 over day 0.

### In vivo proliferation assay

Ten 6-week-old nude mice were injected with TE4^CXCL12+^ (1 × 10^6^) on day 0. Two dorsal skinfold chambers implanted on the back of each mouse. The following day, the animals were assigned to two different groups. The dorsal skinfold chamber was opened, and a mini-osmotic pump (Alzet, 200 μL, pumping rate: 1.0 μL/h) filled with AMD3100 (35 μg/μL) was implanted into five mice; a mini-osmotic pump filled with 0.1 % bovine serum albumin was implanted in five control mice. The pumps were exchanged under general anaesthesia every week. All operations were performed by the same surgeon. All animals underwent measurements of the tumor size on days 3, 7, 10 and 14 to assess whether the blockade of CXCL12-CXCR4 binding inhibits the tumor progression in vivo. One investigator (UY) who was blinded to the treatment measured the tumor size.

### Statistical analyses

Mann-Whitney *U* test, Pearson’s *χ*^2^ test or Fisher’s exact probability test was used to assess the correlation between CXCL12/CXCR4 expression levels and clinicopathological characteristics. The Kaplan-Meier and log-rank tests were used for the survival analysis. Cox proportional hazard models were used for multivariate analysis of variables predicting postoperative survival. In the mouse model and in vitro proliferation assay, the Mann-Whitney *U* test was used to calculate the statistical significance of the tumor size or the absorbance, respectively. All statistical procedures were performed using SPSS v18.0 software (SPSS, Tokyo, Japan). A *p* value of less than 0.05 was considered to be statistically significant.

## Results

### CXCL12/CXCR4 expression and clinicopathological characteristics

**Fig. 1:**
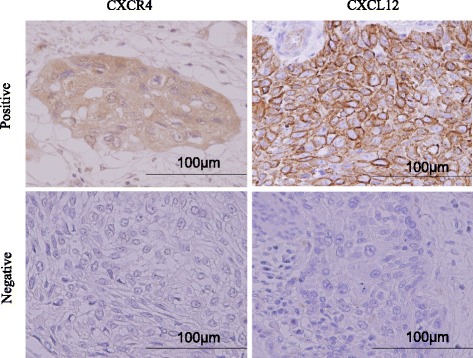
Representative images of immunohistochemical staining of CXCL12 and CXCR4

The patient characteristics are presented in Table 1. The median age of the enrolled patients was 59 years (range: 44–79), and the male/female ratio was 71/8. Representative images of immunohistochemistry are shown in Fig. 1. Forty-eight patients (61 %) were positive for CXCR4 and 62 (78 %) for CXCL12 expression (Table 2). The group with a positive CXCL12 expression was more likely to also have a positive CXCR4 expression compared with the group with negative CXCL12 expression; 41 patients (52 %) were positive for both CXCR4 and CXCL12. We found that patients with a positive expression of CXCL12 tended to have a higher lymph node recurrence rate than other patients (Table 3, *p* = 0.08). No other significant correlation between the expression of CXCR4 or CXCL12 and other clinicopathological factors was found.

**Table 1:** Patient characteristics

	Patients (*n* = 79)
Age (median, range)	59 (44–79)
Gender (male/female)	71/8
Histological grade (1/2/3)	16/57/6
Depth of tumor invasion (Tis/T1/T2/T3)	10/35/7/27
Lymph node metastases (+)	46 (58 %)
Lymphatic invasion (+)	58 (73 %)
Vessel invasion (+)	35 (44 %)
Adjuvant therapy (none/chemotherapy/chemoradiotherapy)	62/16/1
Initial recurrence after surgery (none/lymph node/distant organ/both lymph node and distant organ)	45/14/10/10

**Table 2:** Relationship between CXCR4 and CXCL12 expression

	CXCR4 (−)	CXCR4 (+)	Total
CXCL12 (−)	10 (13 %)	7 (9 %)	17 (22 %)
CXCL12 (+)	21 (27 %)	41 (52 %)	62 (78 %)
Total	31 (39 %)	48 (61 %)	79

*p* = 0.09

**Table 3:** Relationship between expression of CXCL12/CXCR4 and clinicopathological factors

	All patients (*n* = 79) (%)					
	CXCR4(+)	CXCR4(−)	*p*	CXCL12(+)	CXCL12(−)	*p*
	*n* = 48 (61)	*n* = 31 (39)		*n* = 62 (78)	*n* = 17 (22)	
Age, median (range)	58.5 (44–79)	61 (44–74)	0.84^c^	59 (44–79)	57 (50–70)	0.84^c^
Gender						
Male	44	27	0.71^a^	54	17	0.19^a^
Female	4	4		8	0	
Histological grade						
1	11	5	0.69^b^	12	4	0.90^b^
2	34	23		45	12	
3	3	3		5	1	
Pathological T						
Tis	5	5	0.63^b^	8	2	0.39^b^
T1	24	11		28	7	
T2	4	3		7	0	
T3	15	12		19	8	
Pathological N						
Positive	28	18	0.98^b^	36	10	0.96^b^
Negative	20	13		26	7	
Lymphatic invasion						
Absent	13	8	0.90^b^	17	4	1.00^a^
Present	35	23		45	13	
Vessel invasion						
Absent	27	17	0.90^b^	36	8	0.42^b^
Present	21	14		26	9	
Recurrence of lymph node metastasis						
Positive	13	11	0.43^b^	22	2	0.08^a^
Negative	35	20		40	15	
Recurrence of distant organ metastasis						
Positive	13	7	0.65^b^	17	3	0.54^a^
Negative	35	24		45	14	

Recurrence of lymph node metastasis or distant organ metastasis means that the initial recurrence region after ESCC resection was lymph node or distant organ

^a^Fisher’s exact probability test

^b^Pearson’s χ-square test

^c^Mann-Whitney *U* test

**Fig. 2:**
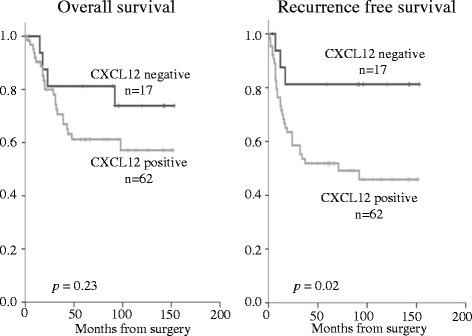
Survival curves of patients after esophagectomy with positive or negative CXCL12 expression. The patients with positive CXCL12 expression exhibit a significantly lower recurrence-free survival (*p* = 0.02)

**Fig. 3:**
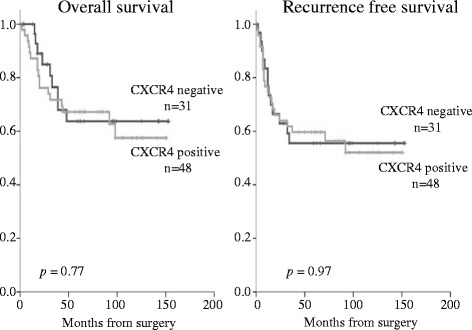
Survival curves of patients after esophagectomy with positive or negative CXCR4 expression. Positive expression of CXCR4 in ESCC is not correlated with survival rate

**Fig. 4:**
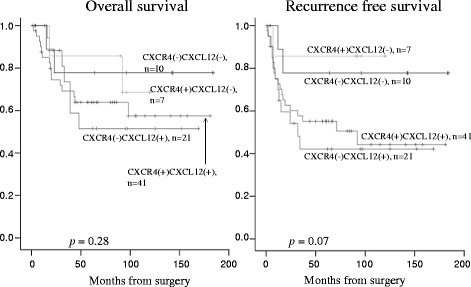
Survival curves of patients after esophagectomy among the four groups. No significant difference was observed; however, CXCL12-positive ESCC tended to be associated with a poor prognosis regardless of the positive or negative expression of CXCR4

The mean follow-up time was 68 months. During follow-up, 34 (43 %) of 79 patients experienced tumor recurrence. Figures 2 and 3 show the Kaplan–Meier curves of recurrence-free survival (RFS) and overall survival according to the expression of CXCL12 or CXCR4. The patients with a positive CXCL12 expression exhibited a significantly lower RFS (*p =* 0.02). CXCR4 expression, however, had no correlation with survival rate. Figure 4 shows the comparison of the survival among the four groups (including CXCL12+ and CXCR4+, CXCL12+ and CXCR4-, CXCL12- and CXCR4-, and CXCL12- and CXCR4+), which showed no significant diffence among them. Patients with CXCL12-positive ESCC tended to have a poor prognosis regardless of the positive or negative expression of CXCR4.

Univariate and multivariate analyses were performed to determine the predictors of subsequent tumor recurrence. We evaluated six variables for survival prognosis by univariate analysis (Table 4). We detected positive CXCL12 expression, lymph node metastasis, lymphatic invasion and vessel invasion as predictive markers of RFS. We performed the multivariate analysis for these four prognostic factors. In conclusion, a positive CXCL12 expression is an independent risk factor for subsequent tumor recurrence, showing the highest hazard ratio compared with other pathological features.

**Table 4:** Univariate and multivariate analyses to determine the risk factor for ESCC recurrence

	Recurrence-free survival		
	Univariate	Multivariate	
Characteristic	*p* value	HR (95 % CI)	*p* value
Positive CXCL12 expression (+/−)	0.021	5.12 (1.54–17.01)	0.008
Positive CXCR4 expression (+/−)	0.97		
pT (≥pT3 vs < pT3)	0.10		
Lymph node metastasis (+/−)	0.005		
Lymphatic invasion (+/−)	0.002	1.84 (1.24–2.72)	0.002
Vessel invasion (+/−)	0.004	2.13 (1.03–4.39)	0.04

*HR* hazard ratio, *CI* confidence interval

We demonstrated subgroup multivariate analysis by dividing the population into two groups by existence of adjuvant therapy. Positive CXCL12 expression was an independent risk factor for postoperative recurrence in the group without adjuvant therapy (*p* = 0.03, hazard ratio = 5.19, *n* = 62). However, no clinicopathological factors, including positive CXCL12 expression were detected as a significant risk factor for recurrence in the group with adjuvant therapy. This was likely due to an insufficient number of patients (*n* = 17).

The MIB-1 proliferation index was markedly higher in ESCC with a positive expression of CXCR4 or CXCL12, especially with positive CXCR4 expression (CXCR4 21.8 vs 7.6 %, *p* = 0.03; CXCL12 17.9 vs 7.6 %, *p* = 0.20; all percentage values are median). As shown in Table 5, ESCC with positive expression of both CXCR4 and CXCL12 exhibited a significantly higher MIB-1 index than the other three groups (24.1 vs 7.5 %, *p* = 0.02). These results suggest that CXCL12 expression increases the proliferation of ESCC through signal transduction after binding to the CXCR4, which in turn leads to an earlier recurrence after surgery. We subsequently surveyed the relationship between CXCL12 expression and ESCC proliferation in vitro and in vivo.

**Table 5:** MIB-1index with or without CXCR4 or CXCL12 expression

	CXCR4 negative	CXCR4 positive
CXCL12 negative	9.6 % (0–58.1 %)	7.2 % (0–52.5 %)
	*n* = 10	*n* = 7
CXCL12 positive	7.2 % (0–51.1 %)	24.1 % (0–68.2 %)
	*n* = 21	*n* = 41

All percentage values are median (range)

*p* = 0.12

### CXCR4 and CXCL12 mRNA expression in TE cell lines

**Fig. 5:**
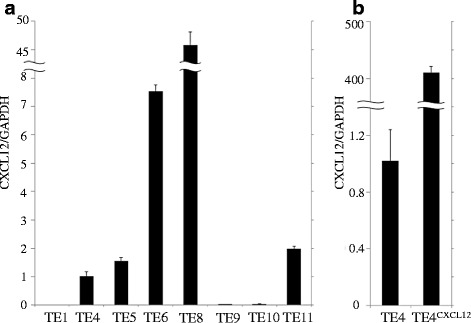
Quantitative real-time RT-PCR (evaluation of CXCL12 expression). **a** The CXCL12 mRNA expression level varies according to the cell lines. **b** The TE4^CXCL12+^ cell line overexpresses CXCL12 410-fold higher than the control TE4 cell line

CXCR4 and CXCL12 mRNA expression in ESCC cell lines were assessed by quantitative real-time RT-PCR. All ESCC cell lines express CXCR4 at different levels (data not shown). Similarly, the CXCL12 mRNA expression level varies among cell lines, with the highest expression level seen in TE8 cells (Fig. 5a) and other cell lines showing a very low expression level or no expression at all. We selected the TE4 cell line for its low expression level of CXCL12 to establish transfectants overexpressing CXCL12. The original TE4 cell line was tumorigenic in nude mice. We established six different TE4^CXCL12+^ cell lines that overexpressed CXCL12 by gene transfer and used one for further investigation. This line was transplantable to mice and showed a 410-fold increase in CXCL12 expression compared with the untreated TE4 line (Fig. 5b).

### In vitro proliferation assay

**Fig. 6:**
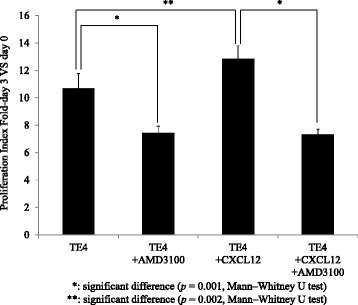
In vitro proliferation assay; TE4 with exposure to CXCL12 and/or AMD3100. TE4 cells exposed to CXCL12 exhibited a significantly higher proliferation index than untreated TE4 cells (*p* = 0.002), and TE4 cells exposed to AMD3100 showed a significantly lower proliferation index than untreated TE4 cells (*p* = 0.001). CXCL12 did not increase the proliferation rate of TE4 cells exposed to AMD3100

**Fig. 7:**
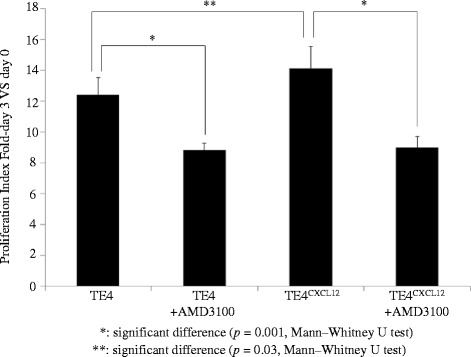
In vitro proliferation assay; TE4 and TE4^CXCL12+^ cells exposed or not to AMD3100. TE4^CXCL12+^ cells showed a significantly higher proliferation index than wild type TE4 cells (*p* = 0.03). AMD3100 significantly decreases the proliferation of both TE4 and TE4^CXCL12+^ cells (both *p* = 0.001). Overexpression of CXCL12 did not increase the proliferation of TE4 exposed to AMD3100

Figure 6 presents the proliferation index of TE4 in the WST cell proliferation assay with or without exposure to CXCL12 and in the presence or absence of AMD3100. When exposed to CXCL12, the TE4 cells showed a significantly higher proliferation index than control (*p* = 0.002). However, when exposed to AMD3100, the TE4 cells showed a significantly lower proliferation index than untreated TE4 cells (*p* = 0.001). CXCL12 was not able to increase the proliferation rate of TE4 cells exposed to AMD3100. Figure 7 shows the proliferation index of TE4 and TE4^CXCL12+^ in the presence or absence of AMD3100. The TE4^CXCL12+^ cells showed a significantly higher proliferation index than the original TE4 cells (*p* = 0.03). Adding AMD3100 significantly decreased the proliferation of both TE4 and TE4^CXCL12+^ cells to the same extent (both *p* = 0.001). The decrease shown in Figs. 6 and 7 was observed not only on day 3, but the tendency was already present on day 1 and 2 (data not shown).

These results indicate that CXCL12 expressed by TE4 or TE4^CXCL12+^ is secreted, binds to its specific receptor, CXCR4, on the cell membrane and stimulates cellular proliferation via signal transduction. AMD3100 inhibits the binding between CXCL12 and CXCR4 and subsequently decreases cell proliferation.

### In vivo proliferation assay

**Fig. 8:**
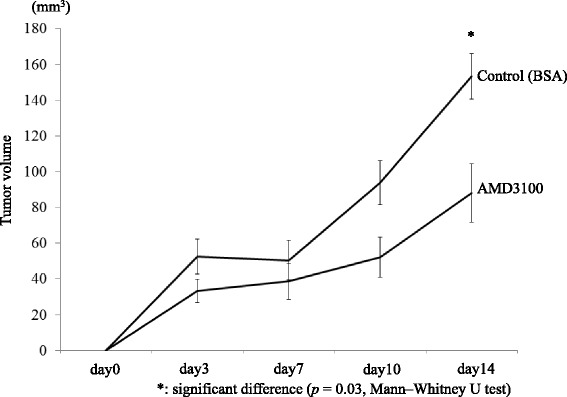
In vivo proliferation assay; TE4^CXCL12+^ with or without infusion of AMD3100. TE4^CXCL12+^ mice continuously infused with AMD3100 showed smaller tumors than control mice at all time points. AMD3100 significantly decreases the tumor size on day 14 (*p* = 0.03)

Figure 8 shows the different tumor sizes following a subcutaneous injection of TE4^CXCL12+^ in the back of nude mice combined or not with a continuous subcutaneous infusion of AMD3100. Mice injected with TE4^CXCL12+^ and infused with AMD3100 had smaller tumors than the controls at all time points. On day 14, the tumor size was significantly decreased by the AMD3100 infusion (*p* = 0.03). This result indicates that AMD3100 decreases ESCC proliferation by inhibiting the CXCL12/CXCR4 signalling pathway artificially enhanced by TE4^CXCL12^. No adverse events were observed during the assay.

## Discussion

Our study on CXCL12, CXCR4 and the progression of ESCC has three significant findings. First, we demonstrate that a positive CXCL12 expression correlates significantly with a lower RFS rate in our patient population with ESCC. However, no significant correlation was detected between CXCL12 or CXCR4 expression levels and any clinicopathological factors, such as T/N stage, vessel or lymphatic invasion. These results suggest that CXCL12 expression in ESCC promotes the proliferation of the tumor and has a direct impact on the recurrence rate, independent of invasion or metastasis. The higher MIB-1 proliferation index with positive CXCL12 expression strongly supports this hypothesis. The multivariate analysis shows that positive CXCL12 expression is the only independent risk factor for postoperative recurrence with a hazard ratio of 5.12, superior to any of the other clinicopathological features. CXCL12 may be a very useful biomarker for predicting the outcome in ESCC patients, and more importantly, could be a critical diagnostic marker for selecting appropriate treatments. In contrast, CXCR4 expression was related to a higher MIB-1 index, but was not significantly correlated with RFS. This indicates that CXCL12 expression has a more prominent role in cellular proliferation, whereas CXCR4 expression only contributes.

Second, we report for the first time that the proliferative abilities of ESCC overexpressing or exposed to CXCL12 are significantly enhanced compared with wild-type cells in vitro. The fact that both CXCL12 overexpression and CXCL12 exposure similarly promote proliferation denotes that ESCC cells expressing CXCL12 secrete this factor. In addition, it also suggests that cellular proliferation is stimulated by the binding of CXCL12 to CXCR4 on the cell membrane. The selective CXCR4 blockade by AMD3100 inhibits proliferation regardless of CXCL12 overexpression or exposure.

Third, AMD3100 inhibits the tumor growth of ESCC expressing CXCL12 in vivo, which has not been previously reported. These results suggest the possibility for autocrine growth in ESCC similar to other carcinomas. Barbieri et al. reported that the overexpression of CXCL12 and CXCR4 induces autocrine and paracrine cellular proliferation in human pituitary adenomas [14]. Moreover, Uchida et al. reported the involvement of an autocrine CXCL12/CXCR4 system on the distant metastasis of human oral squamous cell carcinoma [15]. It is possible that ESCC has an autocrine growth system; however, further studies are required to prove this.

Taken together, CXCL12 is not only a promising biomarker but also a molecular target for inhibiting proliferation. Using CXCL12 as a marker of poor prognosis, patients with CXCL12-positive ESCC are at a high risk of recurrence. Therefore, adjuvant or neo-adjuvant therapy might be indicated to avoid recurrence after an esophagectomy. In terms of molecular therapy, our study denotes the possibility of a cell proliferation blockade through the CXCL12/CXCR4 signalling axis. Our findings provide further evidence for the mechanism of CXCL12/CXCR4-mediated cellular proliferation, leading to novel therapeutic strategies to prevent tumor progression that may bring CXCL12/CXCR4-targeted therapy closer to clinical reality.

However, our immunohistochemical analyses demonstrated that patients with positive CXCL12 expression, including both CXCR4 positive and negative patients, have a poorer prognosis. This result indicates the possibility of additional mechanisms (other than binding to CXCR4 and an enhancement of proliferation) by which CXCL12 promotes ESCC development. Several studies have reported the relationship between CXCL12 and invasion of malignant tumors other than ESCC [18–22]. Kryczek et al. reported that CXCL12 and vascular endothelial growth factor synergistically induce neoangiogenesis in human ovarian cancers [23]. Further studies are necessary to investigate the influence of CXCL12 on tumor cell mortality in the context of ESCC.

Several studies by other groups have demonstrated that the expression of CXCR4 or CXCL12 in cancer cells worsens the prognosis in patients with ESCC. Goto et al. showed that CXCR4 expression is associated with a poor prognosis in patients with ESCC [24], while Sasaki et al. showed that CXCL12 and its receptor CXCR4 correlated with nodal metastasis in submucosal ESCC [25]. Sasaki et al. further reported that positive CXCL12 expression was associated with poor prognosis in patients with ESCC; however, similar to our results, positive CXCR4 expression was not [26]. We thought that this inconsistency was likely caused by the differences in the background of patients. Further assessment regarding CXCR4 expression in ESCC and its function is necessary.

AMD3100 did not fully prevent the proliferation of the TE4 and TE4^CXCL12+^ cell lines in our study, suggesting that CXCL12 may promote the proliferation of ESCC through other pathways in addition to CXCR4. For example, CXCR7, which is a novel receptor of CXCL12, was reported to be involved in the progression of several carcinomas [27–29]. However, we did not assess the expression of CXCR7 in ESCC, and we cannot discuss whether CXCL12-CXCR7 axis is related to the proliferation of the ESCC cell line. Furthermore, our IHC showed that CXCL12 positive expression was related to poor prognosis while CXCR4 positive expression was not. It is possible that this result was caused by CXCR7 involvement (i.e. CXCL12 promotes ESCC progression through not only CXCR4 but also CXCR7, and causes poorer RFS).

Although CXCL12 promoted the proliferation index in vitro, and AMD3100 suppressed the proliferation index in vitro, as well as the tumor size in vivo, our study did not disclose whether the CXCL12/CXCR4 axis is responsible for ESCC survival or apoptosis. Zhou et al. reported that CXCR4 mediates survival of glioma cells through Akt pathway [30]. Moreover, Liao et al. reported that AMD3100 reduces CXCR4-mediated survival and metastasis of osteosarcoma by inhibiting JNK and Akt, but not p38 or Erk1/2, pathways [31]. To assess whether CXCL12/CXCR4 is involved in survival or apoptosis of ESCC, further studies are needed, including an examination of signal transduction following CXCL12-CXCR4 binding.

This study has three other limitations. First, we only surveyed CXCL12 and CXCR4 expression of resected ESCC without preoperative therapy. We cannot conclude that CXCL12 expression is a biomarker of prognosis regardless of chemotherapy or radiation therapy prior to surgery. Second, we used one ESCC cell line, TE4, for in vitro and in vivo studies. TE4 and its transfectant demonstrated that CXCL12 increases cell proliferation; however, we did not test the other cell lines to exclude a cell line specific relationship. Third, we did not evaluate the downstream targets following CXCL12 stimulation. Several studies have reported the involvement of signal transduction, such as the ERK1/2 or Akt pathways in the proliferation of cancers [6, 14]. Therefore, further studies are required to assess the signal transduction after CXCL12-CXCR4 binding in ESCC.

Despite these limitations, our study demonstrates the potential for CXCL12 to become a useful marker for predicting the outcome in patients with ESCC and the development a new therapeutic target molecule to suppress ESCC progression.

## Conclusions

In this study, we demonstrated that the expression of CXCL12 in ESCC is an independent risk factor of recurrence after surgery through immunohistochemistry and we showed that CXCL12 promotes ESCC proliferation. Blocking the binding of CXCL12 to CXCR4 decreases ESCC growth both in vitro and in vivo. Therefore, CXCL12 has the potential to become a biomarker for prognosis prediction and a therapeutic target in patients with ESCC.

## Abbreviations

CXCL12, CXC-chemokine ligand 12; CXCR4, CXC-chemokine receptor 4; ESCC, esophageal squamous cell carcinoma

## References

[1] Jemal A, Siegel R, Xu J, Ward E (2010). Cancer statistics, 2010. CA Cancer J Clin.

[2] Law S, Fok M, Chow S, Chu KM, Wong J (1997). Preoperative chemotherapy versus surgical therapy alone for squamous cell carcinoma of the esophagus: a prospective randomized trial. J Thorac Cardiovasc Surg.

[3] Lee JL, Park SI, Kim SB, Jung HY, Lee GH, Kim JH (2004). A single institutional phase III trial of preoperative chemotherapy with hyperfractionation radiotherapy plus surgery versus surgery alone for resectable esophageal squamous cell carcinoma. Ann Oncol.

[4] Ando N, Kato H, Igaki H, Shinoda M, Ozawa S, Shimizu H (2012). A randomized trial comparing postoperative adjuvant chemotherapy with cisplatin and 5-fluorouracil versus preoperative chemotherapy for localized advanced squamous cell carcinoma of the thoracic esophagus (JCOG9907). Ann Surg Oncol.

[5] Nakamura K, Kato K, Igaki H, Ito Y, Mizusawa J, Ando N (2013). Japan Esophageal Oncology Group/Japan Clinical Oncology Group. Three-arm phase III trial comparing cisplatin plus 5-FU (CF) versus docetaxel, cisplatin plus 5-FU (DCF) versus radiotherapy with CF (CF-RT) as preoperative therapy for locally advanced esophageal cancer (JCOG1109, NExT study). Jpn J Clin Oncol.

[6] Barbero S, Bonavia R, Bajetto A, Porcile C, Pirani P, Ravetti JL (2003). Stromal cell-derived factor 1alpha stimulates human glioblastoma cell growth through the activation of both extracellular signal-regulated kinases 1/2 and Akt. Cancer Res.

[7] Smith MC, Luker KE, Garbow JR, Prior JL, Jackson E, Piwnica-Worms D (2004). CXCR4 regulates growth of both primary and metastatic breast cancer. Cancer Res.

[8] Kollmar O, Rupertus K, Scheuer C, Junker B, Tilton B, Schilling MK (2007). Stromal cell-derived factor-1 promotes cell migration and tumor growth of colorectal metastasis. Neoplasia.

[9] Ehtesham M, Mapara KY, Stevenson CB, Thompson RC (2009). CXCR4 mediates the proliferation of glioblastoma progenitor cells. Cancer Lett.

[10] Irino T, Takeuchi H, Matsuda S, Saikawa Y, Kawakubo H, Wada N (2014). CC-Chemokine receptor CCR7: a key molecule for lymph node metastasis in esophageal squamous cell carcinoma. BMC Cancer.

[11] De Clercq E (2009). The AMD3100 story: The path to the discovery of a stem cell mobilizer (Mozobil). Biochem Pharmacol.

[12] Datema R, Rabin L, Hincenbergs M, Moreno MB, Warren S, Linquist V (1996). Antiviral efficacy in vivo of the anti-human immunodeficiency virus bicyclam SDZ SID 791 (JM 3100), an inhibitor of infectious cell entry. Antimicrob Agents Chemother.

[13] Pawig L, Klasen C, Weber C, Bernhagen J, Noels H (2015). Diversity and inter-connections in the CXCR4 chemokine receptor/ligand family: Molecular perspectives. Front Immunol.

[14] Barbieri F, Bajetto A, Stumm R, Pattarozzi A, Porcile C, Zona G (2008). Overexpression of stromal cell-derived factor 1 and its receptor CXCR4 induces autocrine/paracrine cell proliferation in human pituitary adenomas. Clin Cancer Res.

[15] Uchida D, Onoue T, Tomizuka Y, Begum NM, Miwa Y, Yoshida H (2007). Involvement of an autocrine stromal cell derived factor-1/CXCR4 system on the distant metastasis of human oral squamous cell carcinoma. Mol Cancer Res.

[16] Furusato B, Mohamed A, Uhlén M, Rhim JS (2010). CXCR4 and cancer. Pathol Int.

[17] Uchida D, Onoue T, Kuribayashi N, Tomizuka Y, Tamatani T, Nagai H (2011). Blockade of CXCR4 in oral squamous cell carcinoma inhibits lymph node metastases. Eur J Cancer.

[18] Yadav VR, Sung B, Prasad S, Kannappan R, Cho SG, Liu M (2010). Celastrol suppresses invasion of colon and pancreatic cancer cells through the downregulation of expression of CXCR4 chemokine receptor. J Mol Med (Berl).

[19] Rehman AO, Wang CY (2008). SDF-1alpha promotes invasion of head and neck squamous cell carcinoma by activating NF-kappaB. J Biol Chem.

[20] Wen WW, Xie S, Xin XL, Geng MY, Ding J, Chen Y (2013). Oligomannurarate sulfate inhibits CXCL12/SDF-1-mediated proliferation and invasion of human tumor cells in vitro. Acta Pharmacol Sin.

[21] Shen B, Zheng MQ, Lu JW, Jiang Q, Wang TH, Huang XE (2013). CXCL12-CXCR4 promotes proliferation and invasion of pancreatic cancer cells. Asian Pac J Cancer Prev.

[22] Xue B, Wu W, Huang K, Xie T, Xu X, Zhang H (2013). Stromal cell-derived factor-1 (SDF-1) enhances cells invasion by αvβ6 integrin-mediated signaling in ovarian cancer. Mol Cell Biochem.

[23] Kryczek I, Lange A, Mottram P, Alvarez X, Cheng P, Hogan M (2005). CXCL12 and vascular endothelial growth factor synergistically induce neoangiogenesis in human ovarian cancers. Cancer Res.

[24] Goto M, Yoshida T, Yamamoto Y, Furukita Y, Inoue S, Fujiwara S (2015). CXCR4 expression is associated with poor prognosis in patients with esophageal squamous cell carcinoma. Ann Surg Oncol.

[25] Sasaki K, Natsugoe S, Ishigami S, Matsumoto M, Okumura H, Setoyama T (2008). Expression of CXCL12 and its receptor CXCR4 correlates with lymph node metastasis in submucosal esophageal cancer. J Surg Oncol.

[26] Sasaki K, Natsugoe S, Ishigami S, Matsumoto M, Okumura H, Setoyama T (2009). Expression of CXCL12 and its receptor CXCR4 in esophageal squamous cell carcinoma. Oncol Rep.

[27] Hu SC, Yu HS, Yen FL, Chen GS, Lan CC (2014). CXCR7 expression correlates with tumor depth in cutaneous squamous cell carcinoma skin lesions and promotes tumor cell survival through ERK activation. Exp Dermatol.

[28] Lin L, Han MM, Wang F, Xu LL, Yu HX, Yang PY (2014). CXCR7 stimulates MAPK signaling to regulate hepatocellular carcinoma progression. Cell Death Dis.

[29] Liu Y, Carson-Walter E, Walter KA (2015). Targeting chemokine receptor CXCR7 inhibits glioma cell proliferation and mobility. Anticancer Res.

[30] Zhou Y, Larsen PH, Hao C, Yong VW (2002). CXCR4 is a major chemokine receptor on glioma cells and mediates their survival. J Biol Chem.

[31] Liao YX, Fu ZZ, Zhou CH, Shan LC, Wang ZY, Yin F (2015). AMD3100 reduces CXCR4-mediated survival and metastasis of osteosarcoma by inhibiting JNK and Akt, but not p38 or Erk1/2, pathways in in vitro and mouse experiments. Oncol Rep.

